# ‘Excellence’ and Exclusion: The Individual Costs of Institutional Competitiveness

**DOI:** 10.1007/s11024-016-9298-5

**Published:** 2016-05-04

**Authors:** Richard Watermeyer, Mark Olssen

**Affiliations:** Department of Education, University of Bath, Claverton Down, Bath, BA2 7AY UK; School of Social Sciences, University of Surrey, Guildford, GU2 7XH UK

**Keywords:** Higher education policy, Neoliberalism, Performativity, Audit cultures, Academic exclusion

## Abstract

A performance-based funding system like the United Kingdom’s ‘Research Excellence Framework’ (REF) symbolizes the re-rationalization of higher education according to neoliberal ideology and New Public Management technologies. The REF is also significant for disclosing the kinds of behaviour that characterize universities’ response to government demands for research auditability. In this paper, we consider the casualties of what Henry Giroux (2014) calls “neoliberalism’s war on higher education” or more precisely the deleterious consequences of non-participation in the REF. We also discuss the ways with which higher education’s competition fetish, embodied within the REF, affects the instrumentalization of academic research and the diminution of academic freedom, autonomy and criticality.

## Introduction

In the space of these pages we look at one of the most pronounced and egregious social impacts of what Naidoo (2016) calls the “competition fetish” in UK Higher Education (HE) – academic exclusion. Specifically, we report on evidence of the exclusion of academics by their institutions in the context of research evaluation. We do so to consider the extent to which the neoliberal pursuit of excellence by universities in a market economy of higher education is compromising the personal welfare of academics and constraining, if not condemning, their ability to be creative, critical and, therefore, also competitive knowledge workers. We also consider the extent to which a neoliberal focus on competition is not only causing the fracturing of academic identity but the dissolution of the university as a bastion of free and critical endeavour, and to paraphrase Etienne Wenger (1998), a community working in a unity-of-practice.

Our starting point is a consideration of the university as an institution that has undergone and continues to experience radical organizational and ideological change as a consequence of surrender to neoliberal ideology and New Public Management (NPM) technologies (Couldry 2011; Deem 2004; Docherty 2011; Freedman 2011; Giroux 2007, 2014; Graham 2002; Holmwood 2011; Olssen and Peters 2005; Slaughter and Rhoades 2004). It is concurrently an institution reformed by cognate forces of marketization, massification and globalization. At an ideological level, higher education’s “neoliberalization” (Peck and Tickell 2002) signifies the demise of the traditional Enlightenment idea of the university as a self-managing, collegial and autonomous institution, and the inauguration of a corporate managerial model premised upon a hierarchical, line-management form of governance. In this changing vision of higher education, the university is thus re-appointed as the handmaiden of a global knowledge economy linked to state policies of ‘austerity’ and economic rationalism (cf. Shore 2008).

The shadow, or as we might say, spectre of neoliberalism finds no more emphatic expression than in the pervasive spreading across universities of ‘new managerialism’ (Bok 2003; Deem and Brehony 2005; Deem et al. 2007). This organizational dogma embodies relentless auditing, performance evaluation and benchmarking exercises focused on the quantification of research and teaching excellence manifest in national and international league tables and exemplified, for instance, by the UK’s Research Excellence Framework (REF) and National Student Survey (NSS).^1^ Concomitantly, it is expressed through a culture of quantitative monumentalism or the enactment of what Wernick (1991) calls the “promotional university”.

Where institutions increasingly choose to advertise their rankings not only to the outside world but to their fellow universities (all claiming to be characterized by ‘excellence’), and more invidiously to their own internal academic communities, the consequence is an omnipresence of competitiveness that engenders repulsion, division, discomfort and fear, far more than it might incentivize, harmonize and instill a sense of belonging. As NPM technologies have embedded within the governance of higher education, numerous deleterious and debilitating effects on academic identity and practice have been observed (cf. Deem 2004). For example, direct causality is made between an alleged ubiquity of NPM technologies in HE – and the intensification of new forms of bureaucratization – and the destabilization of academic professional identity (Deem 2008; Henkel 2010; Kolsaker 2013; MacFarlane 2011; Nixon 2008; Slaughter and Leslie 1997; Pick, Teo and Yeung 2012); deskilling (Naidoo and Jamieson 2005); casualization (DiGiacomo 2005); and unbundling^2^ (Kinser 2002).

Concurrently, and despite what appears the increasing fragility, uncertainty and infirmity of academic identity and the academic profession, academics endure ‘more burdensome’ workloads (Corbyn 2009) and unrelenting pressure to succeed in ever more competitive and unforgiving performance-fixated environments (cf. Bazeley 2010; Pollard 2009). Ironically, however, it appears not so much the insecurities of individual academics in response to the inclemency of their new terms of employment, but the performance-related anxiety of institutions that further exacerbates a sense of crisis within the academic profession. Certainly it seems the case that institutional competitiveness intensifies the neoliberal spotlight of surveillance, reflected in the micro-management of academics and also in the transmogrification of the university from free creative space to corporate panopticon. This paternalistic, top-down response to managing competitiveness is counterproductive, however, where it not only erodes academics’ sense of self-efficacy, self-esteem and morale but arrests their capacity to ‘perform’ as creative and critical, even ‘passionate’ knowledge workers (Liu et al. 2011). Furthermore, according to sociologists like Steven Ward (2012), intensive monitoring regimes not only narrow the spectrum of research undertaken – that most likely to receive competitive funding – but can also deflect from core university goals, as staff are required to respond to audit exercises that divert time and attention away from teaching and research.

The most comprehensive, pervasive and arguably pernicious manifestation of academic monitoring in the UK is the performance-based funding system, the Research Excellence Framework (REF) (cf. Collini 2012; Miller and Sabapathy 2011; Murphy and Sage 2014; Sayer 2014), which as a process of research performance evaluation is also the basis of the UK Government’s distribution of Quality Research (QR) monies. It is also the process by which universities are competitively ranked according to their research. The REF, therefore, is a route not only to financial reward but professional esteem, at least for those institutions adept at playing its game. And critically this is what the REF in our analysis is taken to be, a competitive market game. And as in all markets, the key gaming strategies for the institutions involved are to employ a new cadre of managers who become, master tacticians engaged in leveraging high evaluation scores, even where conclusions extrapolated from such scores are only partial and based on incomplete submissions. Furthermore, the REF is a game of increasing stakes that *ups the ante* of performance evaluation.

Unlike its predecessor, the Research Assessment Exercise (RAE),^3^ the REF or more specifically, REF2014, required academics to evidence the societal and economic impact of their research in the form of impact environment descriptions and narrative case studies^4^ (cf. Watermeyer 2012, 2016). The submission of impact case studies, in the context of REF2014, was calculated on the basis of one per ten full-time equivalent (FTE) eligible academics. This metric resulted in institutions limiting the number of academics to be submitted to REF2014 on the basis of proximity to the impact case study threshold (cf. Jump 2015). Plainly speaking, the introduction of impact as a measure of assessment in REF2014 further intensified the selectivity of institutions when nominating who would be entered within their submission and therefore who would be excluded.

It transpires then, that the root of our discussion is an issue of non-representation and the exclusion of considerable numbers of academics, who despite being eligible for consideration in REF2014 were denied admission by their institutions on the basis of their inclusion potentially harming institutional competitiveness: allocation of QR money and superior ranking within research league-tables. We argue herein that REF2014 was utilized more as a form of auditability rather than a means of academic accountability. We claim that this has and continues to cause deep stratification and division across academic cohorts: the continued elevation of an academic elite and oppression of the rank-and-file. While de-professionalizing academics in terms of traditional conceptions of their autonomy, the REF has also contributed to the proliferation of a new professionalized cadre of non-academic managers, with significant power and autonomy (Kolsaker 2013; Sayer 2014). Non-academics have paradoxically become the new power-players within the Academy. The REF is, furthermore, a process, which we argue, has deepened a crisis of legitimacy and self-efficacy for many academics who remain outsiders to its process and perhaps, more worryingly, increasingly their local academic and institutional communities.

Consideration of the sociological impacts of the REF as a distinctive aspect of UK HE governance is especially pertinent, where the REF represents the leading, if not, most high profile and influential performance-based research funding system (PRFS). Indeed, the significance of its study is all the more confirmed by the extent of its imitation by a growing number of HE sectors co-opting PRFS in response to the cautiousness or parsimony of national governments in the distribution of public research monies (cf. Hicks 2012; OECD 2010). Furthermore, while the incorporation of NPM technologies, like the REF, into the governance of higher education is observable across the international HE community, and certainly within the United States, the UK HE sector is distinguished for the speed and/or aggressiveness by which NPM has come to dominate (cf. Deem et al. 2007; Holmwood and Bhambra 2012).

## Method

Our evidence base for this paper comprises REF2014 data published by the UK Higher Education ‘trade paper’, *Times Higher Education*. We consulted this data to identify the extent to which Russell Group^5^ universities as a collective sub-grouping of research-led institutions within the UK’s HE sector, failed to submit members of FTE eligible staff to the REF2014 evaluation process. We also reproduce data relevant to these institutions’ Grade Point Average (GPA)^6^ and GPA weighted with ‘research intensity’^7^ scores to locate divergence and commonalities between these metrics and as a way of illuminating the gamesmanship undertaken by many research-intensive universities in the pursuit of research excellence.

What we have not done nor claim is a sophisticated statistical analysis of evaluation data. This is neither our intention nor interest. Instead, we have made simple calculations from existing data sets related to numbers and percentages of FTE eligible staff submitted to REF2014 to determine the extent of FTE eligible staff *not* submitted. We use the simplicity of these calculations, which are essentially basic subtractions, to evince what we perceive the patently obvious, yet largely ignored mass exclusion and derogation of the academic rank and file.

Finally, it is important to note that whilst the focus of our discussion is primarily with Russell Group institutions as the UK’s research elite, we would not wish to be seen to dismiss similar forms of exclusion brought about by REF2014 and suffered by academics populating non-Russell Group institutions. Furthermore, we recognize that the risk of exclusion through performance evaluation is not only a concern for academic researchers but academic *teachers*, who may be prospectively subjected in the UK to a Teaching Excellence Framework (TEF) (cf. Johnson 2015).

### The ‘University of Excellence’

Writing in 1996, the late Bill Readings spoke of how the emergence of the “university of excellence” challenged the legitimacy of the university and those that worked within it; where Enlightenment grand narratives of truth and freedom had been superseded by a grand narrative of the market. Other commentators, like Jon Nixon (2008), have similarly bemoaned a state of crisis in the university where a struggle for competitive advantage between higher education institutions in national and international contexts has caused the corrosion and increasing evisceration of the idea of the university as a space of cultural and scientific critique and creativity. The marketization of higher education and an increased emphasis for universities to rationalize their activity and justify their public patronage on the terms of their economic and societal contributions, calls for a new kind of academic or academic specialization. Academics predisposed to performing in innovative and entrepreneurial ways are those most able to provide authoritative and compelling accounts of the ways with which they are excellent^8^. These are, to borrow from Becher and Trowler (2001) a new “tribe and territory” of academics, more market conscious and savvy, more flexible in terms of adapting their identity and practice to a market logic, or alternatively more cognizant of their vulnerability in the context of a precarious and unforgiving labour market. These are academics that arguably are rated not for the excellence of teaching or research but their excellence of administration (Rolfe 2013). Beyond this cohort of academic entrepreneurs and largely capitalist elites lies a significant population at risk (cf. Jump 2014), unable, or perhaps unwilling, to comply, or in many cases gain equal entry and license to participate, with the standards and protocol demanded by the university of excellence.

### Evaluating Excellence: Excluding Researchers

The attribution of excellence as a value is problematic because its meaning is polysemic; without consensus; and susceptible to producing radical uncertainty. More troubling is that beyond conceptual variance and disparateness is a misassumption that excellence is an achievement or goal when it is instead a largely redundant and meaningless qualifier (Rolfe 2013). Indeed, in our reckoning, an aspiration of excellence seems as much amorphous as futile; a truly Sisyphean pursuit. This is, we rationalize, why, where a universal qualification of excellence is elusive, its quantification assumes precedence. The quantification of excellence provides a way out of the incertitude of its qualification and according to NPM proselytes, enables articulation and measurement of efficiency and profitability. It is ostensibly, therefore, well purposed to ‘describe’^9^ a market economy of knowledge and education. In our estimation, however, a shift towards the quantification of excellence provides no greater advantage than a U-turn into a *cul-de-sac*.

Significantly, not all research active staff were submitted by their institutions for consideration in REF2014. Instead, researchers were selected or ‘cherry-picked’ by institutional managers on the basis of ‘calculations’^10^ or rather, safe-bets that their research would return high evaluation scores. Determining REF eligibility or ‘returnability’^11^ necessitated most institutions administering an internal peer-review process that preceded the REF’s own ‘independent’ review of publications and impact case studies. The results of REF2014 confirmed considerable variation in the numbers of eligible staff submitted by their universities and an opportunity for universal submission being bypassed by institutional strategists in favour of ‘playing-the-game’ of Grade Points Average (GPA). Whilst GPA in simple terms is designed to reflect the ‘excellence’ of research outputs and impacts submitted for evaluation and comprises, therefore, the main indicator of the quality of research within an institution, large numbers of outputs and impacts that might have been considered within REF2014 were ignored and were failed to be included in many universities’ submissions. For institutions failing to return a majority of eligible academic staff, their research intensity, which reflects the depth of research quality across a whole institution, lessened. In some instances, the gap between GPA and research intensity was pronounced and provided firm indication of institutions playing the game, albeit at the expense of large numbers of academic staff.

When the initial REF2014 results were published, the trade press such as the *Times Higher Education* (THE) was without access to data, subsequently provided by the UK’s Higher Education Statistics Agency (HESA), covering numbers of staff returned against those not by institutions. An initial organization of scores by GPA reported in THE revealed a top ten populated in descending order by the Institute of Cancer Research, Imperial College London, London School of Economics and Political Science, University of Oxford, University of Cambridge, Cardiff University, King’s College London, University College London, University of Warwick and the London School of Hygiene and Tropical Medicine. However, only a week later when research intensity data was made available, THE published a new set of results and league positions that incorporated and were weighted according to research intensity. This altered the picture somewhat, with some new entrants to the top ten, some dropping out, and an overall reordering for those that remained. Most dramatically, one institution, Cardiff University, moved from a position of 6th in the initial GPA rankings to 50^th^ when its submission was recalculated according to research intensity.

Of course, league tables may be interpreted and reported by their stakeholders in a variety of ways and there is perhaps no clear sense of which constitutive data set is most reliable or offers the most authoritative, complete or honest declaration of excellence. Notwithstanding, institutional claims of excellence may only be considered partial where attribution is made not to the entirety of an academic body but sub-set of it. The case of Cardiff University in REF2014 provides one such example of selective interpretation where institutional excellence was claimed but only linked to less than two-thirds (or 62%) of its eligible staff. What then for the remaining 38% perceived unfit to represent the university? Cardiff University was not alone in such game-playing. Other universities, particularly in Wales, were guilty of returning far fewer in REF2014. Neighbouring institutions such as the University of South Wales returned only 714 or 16% of its eligible staff, while Cardiff Metropolitan University returned a meagre 381 or 9% of its eligible staff. The extreme paucity of participation by academics from both of these institutions in REF2014 reflects an even more aggressive policy of selectivity operating within newer and less research-intensive, non-Russell Group institutions^12^, which necessitates further scrutiny. Slightly bucking the trend, Swansea University, as Wales’ second-highest ranking institution in both GPA and research intensity frameworks, submitted 71% of its eligible staff, moving it in the latter framework eight places above Cardiff University to 42nd.

From a sociological perspective, the most alarming yet less reported aspect of research evaluation is the failure to universalize the research evaluation process and the blatantly, exclusionary policies pursued by some institutions. When we look across the UK’s Russell Group institutions, the purported heartland of the UK’s research excellence, the cumulative figures are even more troubling. In Table 1 we provide an alternative ranking for the REF based on the number of eligible staff excluded from the evaluation process.

**Table 1:** Russell Group REF2014 Scores by *Non-submission*

Institution	GPA Ranking	Research Intensity weighted GPA Ranking	Number of FTE staff submitted	Number of FTE staff eligible	% & Number of eligible FTE staff *not submitted*	***Non-submission*** **of FTE eligible staff ranking**
Cardiff University	6	50	738	1,182	38% n=444	**1**
University of Liverpool	33	46	760	1,083	30% n=323	**2**
Queen Mary University of London	11	34	671	911	26% n=240	**=3**
University of Sheffield	14	33	1,043	1,405	26% n=362	**=3**
University of Leeds	21	34	1,149	1,535	25% n=386	**=4**
University of York	14	32	643	862	25% n=219	**=4**
University of Manchester	17	26	1,561	2,002	22% n=441	**5**
Durham University	20	24	740	940	21% n=200	**=6**
University of Nottingham	26	28	1,404	1,778	21% n=374	**=6**
King’s College London	7	17	1,369	1,718	20% n=349	**=7**
Newcastle University	26	26	888	1,115	20% n=227	**=7**
University of Birmingham	31	23	1,065	1,320	19% n= 255	**8**
University of Exeter	30	19	736	900	18% n=164	**9**
University of Edinburgh	11	12	1,753	2,105	17% n=352	**=10**
University of Warwick	8	11	931	1,115	17% n=184	**=10**
University of Glasgow	24	15	1,099	1,312	16% n=213	**11**
London School of Economics and Political Science	3	7	532	628	15% n=96	**12**
University of Oxford	4	5	2,409	2,775	13% n=366	**13**
University of Southampton	18	8	1,113	1,240	10% n=127	**14**
University of Bristol	11	5	1,138	1,246	9% n=108	**=15**
University College London	8	4	2,566	2,810	9% n=244	**=15**
Imperial College London	2	3	1,257	1,368	8% n=111	**16**
University of Cambridge	5	2	2,088	2,196	5% n=81	**=17**
Queen’s University Belfast	42	8	868	916	5% n=48	**=17**

An examination of the figures published by THE reveal not only significant variance between the ranking position of institutions by GPA and intensity weighted GPA but a significant number of eligible academics not returned by Russell Group institutions. Our calculations are that while, 28,251 FTE eligible staff were submitted across the Russell Group, 5,941 FTE eligible staff were not; which corresponds to 21% of Russell Group ‘eligible’ academics not featuring within REF2014. From another perspective, the number of eligible ‘Russell Group’ academics not participating in REF2014 would be greater than the combined submissions of Cambridge, Oxford and King’s College London. Whilst this in itself is something of a hyperbolic analogy, it is nevertheless a calculation that disrupts the claims of many Russell Group institutions to research greatness.

In our alternative ranking of Russell Group institutions not by GPA or research intensity weighted GPA but number of staff not participating in REF2014, Cardiff University leads the way while the Universities of Cambridge and Queen’s University Belfast in joint last are arguably the most inclusive of the Russell Group institutions, both omitting ‘only’ 5% each of their eligible staff. Nonetheless the entirety of the Russell Group fraternity appears to claim the same kind of research excellence. How can this be the case? While the claims of Cambridge appear strongest where the percentage representation of staff and concurrently high GPA and intensity weighted scores evidence slight fluctuation, divergence between GPA and intensity weighted GPA by and across institutions suggests that institutional claims of research evidence are rather ambiguous. Furthermore, without universal submission, whole-institutional claims of research excellence are rendered less than persuasive. Ultimately, what these configurations and various value matrices demonstrate is not only elasticity in the interpretation and application of REF2014 results, but that as an evaluation exercise, issues of comparability and, therefore, ranking were fraught by gulfs in the institutional treatment of academics as either essential or expendable. Furthermore, these results may be interpreted as revealing, at either end, the extent to which institutions’ embraced either a model of neoliberal managerialism or collegial democracy.

While many institutions claimed success and evidence of their competitiveness through REF2014, there exist reports of individuals whose exclusion from it has resulted not only in a sense of professional failure and degradation but fear of and subjection to intimidation and bullying. Even before the publication of REF2014 results, a poll conducted by the UK’s *Guardian* newspaper into work-place bullying revealed that 73% of a cohort of 1,366 respondents felt that the REF was further contributing to a bullying culture in UK universities. This figure rose to 81% among those working in Russell Group institutions and further still among Researcher (85%) and PhD candidates (91%). The UK University and College Union^13^ (UCU) (2013) also reported on the particular challenge of the REF for early career academics and its influence in accentuating requirements for entry-level academic positions; predominantly a demand for applicants to demonstrate four ‘returnable’ or ‘REF-able’ outputs (Bishop 2015). This kind of criterion, however, we would suggest, is an unreasonable if not entirely unrealistic expectation where many, if not most early career academics will suffer the precariousness of insecure and short-term appointments that do not permit independent research and may, furthermore, result in applicants deviating from and abandoning the focus of their doctoral work. Only a few benefit from funded post-doctoral fellowships that provide time and finance with which to translate doctoral theses into the pre-requisite quartet of outputs. Yet even then, ‘returnability’ is far from guaranteed where the quality threshold demanded by the REF is dizzying and where only outputs considered three to four star quality attract Government remuneration. To provide greater context to this, outputs considered to be of two-star quality, defined in REF2014 as “quality that is recognized internationally in terms of originality, significance and rigour” (www.ref.ac.uk), would not have been considered ‘REF-able’ and, therefore, would likely have been excluded from institutional submissions. So, in other words, in the terms of REF2014, to conduct research that is internationally recognized is to fall some way short. Taking this a step further, four-star quality, which was defined as “quality that is world-leading in terms of originality, significance and rigour” (ibid.), would demand in all probability significant cash flow and ability on the part of researchers to leverage-in large research funds. The likelihood of this eventuality among academics just starting out and without the necessary reputational capital is, even in the most optimistic prognosis, slim.

The path to ‘returnability’ would appear to be made all the more perilous where institutional REF administration and administrators are considered by some to be respectively, inconsistent^14^ and lubricious. UCU (2013a), for instance, has reported “a number of deficiencies in institutional procedures established to select which outputs and individual researchers [were] submitted to the REF”. UCU reported skepticism apropos the competency and neutrality of REF managers and reviewers; complaints of a lack of transparency; and intentional obfuscation in communicating, if at all, the justification for academics’ inclusion or exclusion within institutional REF2014 submissions; and the use of journal rankings in deciding whether to include outputs even though doing so contradicts assurances made by funding councils that such rankings would not be used as an evaluative criterion. More troubling, the UCU (ibid.) also reported on grievous malpractice in universities and threats made by some institutions of punitive action for academics that were not included in their REF2014 submissions. Threats reported (UCU 2013b) include academics having research privileges rescinded; contracts redrawn to ‘teaching-focused’; and being placed under ‘capability’ measures^15^. The implication of these threats is not only that the value of teaching is undermined but that academics’ career prospects are irreversibly damaged (cf. Fazackerley 2013). Furthermore, maligning university teachers as second-class citizens instead of treating them in a parity of esteem with researchers, is as Kelly (2013) argues, foolish not only on ideological but pragmatic grounds where the student marketplace is increasingly competitive and nevermore so important to universities’ solvency.

### Evaluating Excellence: Excluding Research

At the same time that researchers are being excluded by the competition fetish, research agendas are increasingly streamlined, instrumentalized and adapted to fit the strategic priorities of research councils (Burawoy 2011; Docherty 2011). The organization of research by researchers – and top-down by research managers and administrators – is also now much more strategic and designed to follow the timeframes and requirements imposed by evaluation systems like the REF. For instance, academics are now inclined, or more realistically, compelled, to organize their research activity into the window of time in-between REFs, typically four-to-five years. This time-period represents the opportunity with which to conduct research; organize for its impact; and ensure its culmination with at least four ‘excellent’ outputs necessary for consideration within a university’s submission. In no shape or form is any account made for the perambulatory and serendipitous nature of research as a process of discovery.

Where academics appear increasingly to follow a ‘4 × 4’ route to research excellence – four ‘outstanding’^16^ publications within a four-year period – the production of knowledge appears increasingly constrained by the process of its evaluation. Indeed, the terms of evaluation appear increasingly to dictate the ways with which academics conduct research (cf. Moriarty 2011). Some academic managers, for instance, now argue that in responding to the terms of research evaluation, when a book has equal weighting with a journal publication, academics should abandon the first and focus on the latter because the cost-benefit ratio is more favourable. We also have experience of institutional managers who argue that academics should only write-up research which is the recipient of external funding, as if funding is the only barometer of the merit and quality of research. In this context, this very article, much as a multitude of book chapters would be no longer possible. What also of ‘research’, which requires no funding and does not feature as a Research Councils, UK (RCUK) funded work-package, but is the consequence of curiosity and blue-skies thinking, and for research which is unlikely to attract funding, not because of any concern regarding quality or value, but because it does not conform to the strategic plans and prioritizations of research funders and their reviewers?

In the era of what Gornall and Salisbury (2012) term academic ‘hyperprofessionality’, the extent to which academics might return on a 4x4 model is compromised by the various and arguably excessive demands of mechanisms such as work allocation models and key performance indicators. Where such managerial devices are intended to increase the productivity, profitability and efficiency of academic staff, they are resisted and bemoaned for the extent to which they unnecessarily obfuscate and impede academics’ attempts to perform as teachers and researchers. They are also responsible for ‘burn-out’ and attrition, as academics leave the Academy no longer able to cope or deliver on institutional demands (cf. Warner 2013). Work allocation models and key performance indicators are another manifestation of the quantification of excellence, which appears, contrary and antagonistic to the terms of academic endeavour, when grounded and led by creativity. The new terms of competition in HE, informed by its marketization and a surrender to neoliberal axioms or what Michael Burawoy (2011) refers to as the imposition of “Soviet planning” are arguably, therefore, responsible for the denouement of academic creativity, reflexive knowledge, criticality and innovation. They are certainly entirely at odds with traditional liberal and pre-liberal models of academic professionalism and Merton (1942) norms of universalism, communalism, disinterestedness and skepticism.

The ascent of managerialist cultures in HE and the growing ubiquity of performance-metrics into every crevice of academic life is blamed for the erosion of creative flair and to borrow from Allan Bloom (1987), the closing of the intellectual mind and degeneration of the university as a knowledge incubator. Whilst competition, if proportionate or rather non-excessive, may inspire creative thinking, in the current HE landscape it features not as a catalyst of creativity but conformity. Competition in this context is not implemented for the purpose of challenging the limits of academic imaginations and adventures but in constraining these to a uniform expression of what counts. Any idea of academia as a portfolio of diverse undertakings would seem to be excluded *tout court*. In addition, as we have already established, demonstrating what counts and moreover what counts as excellence is an entirely arbitrary process that is prone to radical uncertainty and polarized opinion (cf. Lamont 2009). Yet the way with which knowledge is evaluated and excellence attributed is entirely dependent upon academics forcing knowledge into physical and measurable manifestations or in REF parlance, units-of-assessment, which academics attempt to satisfy through research outputs and impacts.

To our mind, a belief that research can be unproblematically converted into a product that can be measured and controlled in purely market terms is false. Market competition is necessarily zero-sum and produces an endless spiral of winners and losers. Of course, research is not a market product in this sense. It is a human process. While some sensible absolute criterion of accountability is clearly important in order to establish reasonable minimum standards, and ensure a fair days work for a fair days pay (as it were), it would seem hardly necessary to colonize the entire academic research agenda organized in five/six-year cycles via a bureaucratic revolution of nightmarish proportions for this modest goal to be achieved. One could be forgiven for thinking that the initial audit trail, initiated by Thatcher in 1986, drawing on a library of neoliberal thinkers, contained within its rationale, a deep distrust of academics and a pervasive anti-intellectualism. If evaluation of research should be focused on anything, it might be in determining how academics can improve a process that is constantly evolving, instead of providing a false sense of accomplishment.

The compartmentalization of research into a 4 × 4 framework, and similarly the modularization and slenderization of university education (cf. McGettigan 2013), suggests that knowledge may be contained and stored in neat boxes. It also intimates ownership and a sense that academic outputs are an individual or institution’s property, where it might be more helpful and profitable to think of ideas not as academic capital, but what Hannah Arendt (1958) calls, a means for “public action”. Surely then, as Cooper (2015) argues, this orientation provides a more meaningful basis from which to organize and achieve academic visibility and impact. Regrettably, however, the most profound contradiction of impact evaluation in the REF is that it actually curtails the possibility for ideas to translate into public action, where it restricts the kinds of conversations and collaborations between academics and the publics to those that might more easily be claimed to have impact.

For all the investment in UK HE in knowledge exchange and transfer (cf. Watermeyer 2011, 2016) there appears an explicit bias towards knowledge not for social action but for either commercial exploitation or what could be termed as functionalist societal accommodation. In other words, the REF as presently constituted, would appear to incorporate those exemplars that enable universities to include what they perceive to be their strongest, most marketable impact case studies. This, it can be argued cogently, is likely to be the outcome of a preparatory process internal to individual universities where the academic promotion by departments or individuals of impact case studies and other auditable content must necessarily compete for inclusion. The kinds of public or community engagement that generate ‘soft’ impact which are less well-represented in the REF are those at risk, when they are not only not endorsed but actively discouraged by senior academic and institutional managers (cf. Watermeyer 2015). The Bunyanesque shadow of hard impacts, those engineered through tightly defined and regulated *stakeholder* interactions, may likely relegate the merits of *public* interactions to obscurity. In a race to embrace the triple-helix (cf. Leydesdorff and Etzkowitz 1996), universities seem to be denying academics the opportunity with which to engage a broader public in a genuinely critical sense. Concurrently, notions of a ‘quadruple helix’, which might incorporate the public as an integral member of the knowledge society, seem, at least for the immediate future, fantastical.

## Conclusion

As the consummate manifestation of the competition fetish, the REF is a force with potentially disastrous consequences for the future of the academic profession and scientific endeavor more generally. Where the number of academics being excluded from the process of research evaluation is so vast as to effectively dismiss the research contribution of three major global universities, there must be questions asked of the legitimacy of the research evaluation process; the claims of research excellence made by universities; and the quality and extent of support provided by universities in enabling the research endeavors of their academic staff. Furthermore, the political and academic elites responsible for the REF surely must be forced to heed the call of those who declaim its credibility as a sound indicator of the quality of academic research (cf. Copeland 2013) and mechanism for improving the UK’s research contribution (UCU 2013c).

The presidential incumbents of the British Academy and Royal Society recently stated that, “If the present system is not encouraging researchers and scholars to pursue the best, most profound and most important lines of research, then we need to create one for 2020^17^ that does” (Stern and Nurse 2014). Our analysis, similar to that posted by the Council for the Defence of British Universities (2015), reveals that the system is certainly not working and requires change before more damage is done to academic identity and practice. The first change must be that academics are treated in a parity of esteem and made eligible in a universal system of assessment. Assessment should then be focused not on auditability but accountability and on satisfying the potential of science, less the demands of those who try to rule it. The REF has not to date but must, if it is to have a future, “ensure fair play for all” (Gill 2012).

The materialization of a democratically informed and operationalized alternative to the REF – one that might avert competitive factionalism and facilitate an equal and harmonious comm*unity*-of-practice – necessitates consultation with the *entire* membership of UK HE – an event not yet realised, perhaps currently unimaginable, and an outcome of which academics might only speculate upon. The challenge then for the UK (and international) HE community is to reach beyond a fixation with *scoring* research and researchers and embrace a far more meaningful approach to realising the creative and critical potential of its academic flock. To achieve this it will be necessary to substantially blunt the edge of managerialism through serious structural administrative and pedagogical reforms that will in turn re-professionalise academics and re-empower them within their universities and within academia more generally. This will involve a number of organizational and administrative changes that can only be alluded to here – including perhaps reinstating the power of Senates and reappointing academics to Councils – which might reverse the process suggested by McCormick and Meiners (1988) in America, and implemented by universities across the developed world, to appoint greater numbers of business and ‘lay’ advisors to Councils, as well as disestablishing closed executive boards appointed by Vice-Chancellors. These and other reforms are now needed in order to restore academics as the rightful custodians of the academic estate.

## Footnotes

1 Changes in UK Government policy concerning undergraduate admissions in UK universities – specifically the removal of a cap on student numbers and raising of a tuition fee cap – have further intensified the competitiveness of a higher education market economy and concurrently the significance of league table as a consumer guide used by students, influencing their choice of where to study.

2 ‘Unbundling’ refers to the disaggregation or sub-division of academic work into specialist functions.

3 The RAE was the first iteration of a UK system of national research evaluation begun in 1986 and repeated on four subsequent occasions until the last in 2008, after which it was replaced by the REF, the results of which were published in December 2014 – hence REF2014.

4 ‘Impact’ in REF2014 accounted for 20% of the total evaluation, which also included ‘research outputs’ (65%) and ‘research environment’ (15%).

5 ‘The Russell Group represents 24 leading UK universities which are committed to maintaining the very best research, an outstanding teaching and learning experience and unrivalled links with business and the public sector’ (www.russellgroup.ac.uk).

6 Grade Point Average (GPA) is a measure of the overall or average quality of research, which disregards information related to the percentage of an institution’s FTE eligible staff.

7 Research Intensity is a measure calculated by multiplying an institution’s GPA score by the percentage of its FTE eligible staff.

8 And as a new component of research evaluation in the UK, ‘impactful’ where academics are charged to evidence the societal and economic impact of their research through narrative case studies.

9 We perceive numerical description as largely meaningless without qualitative contextualization. The quantification of excellence is whilst compliant with the needs of administration no more helpful in eliciting the value of research. Indeed, such valuation holds even greater scope for misappropriation, not least where readers of numerical values are prone to quantitative mesmerisation.

10 Internal peer-review itself was problematized by a sense of staring into the abyss and second-guessing what REF panellists would register as excellence. In the context of impact evaluation in REF2014, determinations and/or definitions of excellence were seen to be especially hard for researchers to predict.

11 ‘Returnability’ or capacity to ‘be returned’ refers to the extent to which a researcher satisfies the conditions of being entered into the REF and broadly translates into having produced four outputs considered to be of four-star quality.

12 In data published in the *Times Higher Education* (January 2015), institutions in the lowest percentile of eligible staff submitted in REF2014 were all ‘new’ universities. Southampton Solent University was the lowest scoring institution, submitting only 7% of its eligible staff.

13 The UCU is the largest post-school union in the world with a membership of 120,000 comprising academics, lecturers, trainers, instructors, researchers, managers, administrators, computer staff, librarians and post-graduates working across UK based universities, colleges, prisons, adult education and training organizations. For further information: www.ucu.org.uk.

14 Inconsistencies in the official REF peer-review process are also reported by Holmwood and McKay (2015).

15 HESA (2014) statistics, (published 18th November 2014) reveal a 2.1% increase in the numbers of university staff on a teaching-only contract between December 2012–2013, or in other words, as Grove (2014) reports, a significant rise in teaching-only contracts in the REF year.

16 ‘Outstanding’ being as much an ambiguous and subjective qualifier as ‘excellent’.

17 Prospective date of the next REF.
